# Synergistic Effect of Solvent Vapor Annealing and Chemical Doping for Achieving High-Performance Organic Field-Effect Transistors with Ideal Electrical Characteristics

**DOI:** 10.1021/acsami.2c16760

**Published:** 2023-01-18

**Authors:** Jinghai Li, Adara Babuji, Lamiaa Fijahi, Ann Maria James, Roland Resel, Tommaso Salzillo, Raphael Pfattner, Carmen Ocal, Esther Barrena, Marta Mas-Torrent

**Affiliations:** † Institut de Ciència de Materials de Barcelona, ICMAB-CSIC, Campus UAB, 08193Bellaterra, Spain; ‡ Institute of Solid State Physics, Graz University of Technology, Petersgasse 16, Graz8010, Austria; § Dipartimento di Chimica Industriale “Toso Montanari”, University of Bologna, Viale del Risorgimento 4, 40136Bologna, Italy

**Keywords:** OFETs, doping, organic semiconductors, solvent annealing, contact resistance, interfacial traps

## Abstract

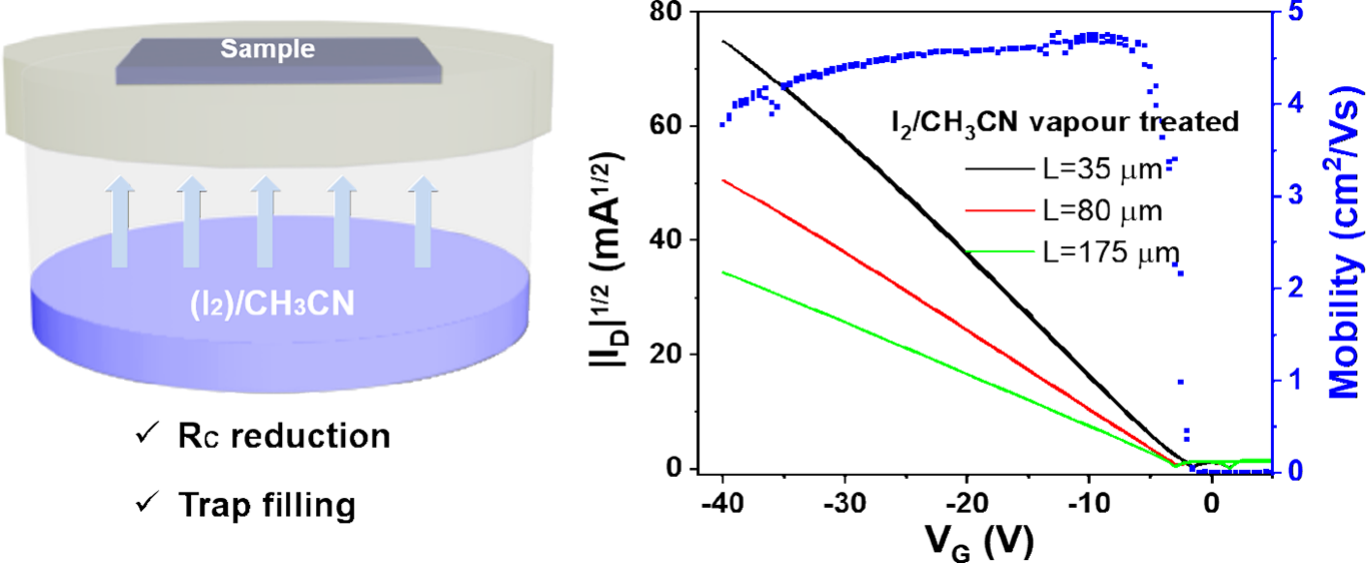

Contact resistance and charge trapping are two key obstacles, often intertwined, that negatively impact on the performance of organic field-effect transistors (OFETs) by reducing the overall device mobility and provoking a nonideal behavior. Here, we expose organic semiconductor (OSC) thin films based on blends of 2,7-dioctyl[1]benzothieno[3,2-*b*][1]benzothiophene (C8-BTBT-C8) with polystyrene (PS) to (i) a CH_3_CN vapor annealing process, (ii) a doping I_2_/water procedure, and (iii) vapors of I_2_/CH_3_CN to simultaneously dope and anneal the films. After careful analysis of the OFET electrical characteristics and by performing local Kelvin probe force microscopy studies, we found that the vapor annealing process predominantly reduces interfacial shallow traps, while the chemical doping of the OSC film is responsible for the diminishment of deeper traps and promoting a significant reduction of the contact resistance. Remarkably, the devices treated with I_2_/CH_3_CN reveal ideal electrical characteristics with a low level of shallow/deep traps and a very high and almost gate-independent mobility. Hence, this work demonstrates the promising synergistic effects of performing simultaneously a solvent vapor annealing and doping procedure, which can lead to trap-free OSC films with negligible contact resistance problems.

## 1 Introduction

The success in improving charge carrier mobility in organic field-effect transistors (OFETs) added to the development of high-throughput processing techniques have strongly relaunched the interest and increased the perspectives of these devices in applications.^1−5^ However, OFETs still suffer from some electrical nonidealities, which represent clear bottlenecks for their practical implementation.^6^

Contact resistance (*R*_c_) in OFETs is a serious limiting factor in the development of high-performance organic devices.^7−10^ Contact resistance is caused by the misalignment between the metal work function and the energy levels of the OSC and can result in devices electrically dominated by the injection of charges rather than by the charge transport through the OSC. Devices with high *R*_c_ can exhibit a high dependence of the mobility on the gate voltage and, therefore, the mobility can be dramatically overestimated.^11,12^ Further, it has been reported that the development of high-frequency applications based on OFETs critically relies on finding routes to significantly reduce *R*_c_.^13−16^

Charge trapping is another source responsible for device deficiencies.^17^ Electronic states in the energy gap of OSCs that can come from structural defects, chemical impurities, and environmental effects can trap charge carriers.^18−20^ Depending on their relative energetic position with respect to the band edge, deep or shallow traps are created. Although the presence of charge traps is one of the major causes that deviates devices from an ideal behavior, the preparation of trap-free OSC thin films, especially when employing printing techniques, is extremely challenging. When the gate voltage is applied in an OFET, first, the traps have to be filled before the device is switched on. Hence, a high density of charge traps has a detrimental impact on the device mobility, leads to gate-dependent mobility and to an increase in the threshold voltage, and hinders the bias stress stability.^6,21^

Considering all the above, it is clear that charge trapping and contact resistance are two key obstacles, often intertwined, that negatively impact on the OFET performance. Chemical doping has recently been considered as a promising key enabler for improving device performance.^22^ Doping has mainly been exploited to increase device mobility, adjust the threshold voltage, fill up trap states, enchance the stability with time, or improve charge injection by contact doping.^18,23,24^ Despite the latest encouraging results, there is still a limited number of established doping protocols.^25,26^ Moreover, solvent vapor annealing has also been shown to be an excellent tool to improve the OSC film quality, mainly in terms of thin film crystallinity,^27^ but it can also be applied for reducing the density of charge traps.^28^

Here, we employed a new strategy to greatly improve the OFET performance by exploiting the synergistic effect of solvent vapor annealing and chemical doping. By performing a careful comparitive study, we prove that while the vapor annealing process reduces shallow traps in the films, the chemical doping of the OSC film is responsible for the diminishment of deeper interfacial traps. As a result, devices with ideal characteristics are achieved, which exhibit an enhanced and almost gate independent mobility and a reduced contact resistance.

## 2 Results and Discussion

**Figure 1:**
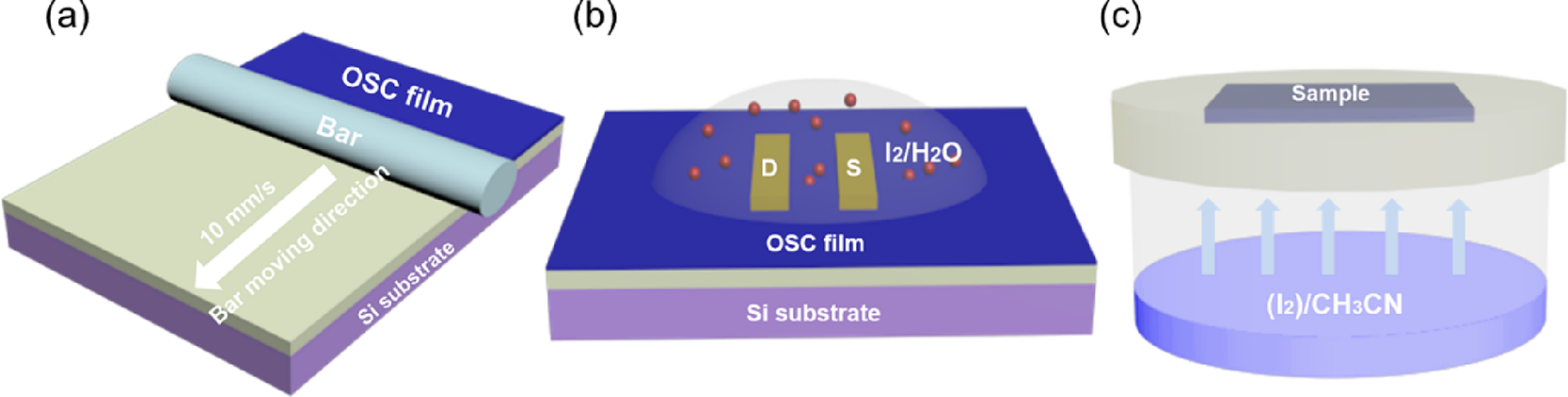
(a) Schematic illustration of the BAMS technique. Scheme of the OSC thin film treatments: (b) I_2_/H_2_O treatment and (c) exposition to CH_3_CN or I_2_/CH_3_CN vapors.

Thin films of blends of 2,7-dioctyl[1]benzothieno[3,2-*b*][1]benzothiophene (C8-BTBT-C8) and polystyrene (PS) in a mass ratio of 4:1 were prepared on SiO_2_ substrates employing the bar-assisted meniscus shearing technique (BAMS, Figure 1a), as previously reported.^3,29^ The deposition of these blends by BAMS results in a vertical phase separation of the two materials, where a crystalline layer of the C8-BTBT-C8 semiconductor sits on top of a PS layer that is in contact with the SiO_2_ substrate.^3,26,30^ Gold source-drain top contacts were thermally evaporated through a shadow mask consisting of device motifs with identical channel widths (*W*) but with different channel lengths (*L*) (see Experimental Section).

Four sets of devices were prepared: (1) *untreated*, or also called *pristine*, *devices*; (2) *vapor-annealed devices*, exposed to acetonitrile (CH_3_CN) vapors; (3) *chemical-doped devices*, treated with an aqueous iodine solution (I_2_/water); and (4) *vapor-annealed and chemical-doped devices*, exposed to vapors of an I_2_/CH_3_CN solution (Figure 1b, and see Experimental Section for further details). It should be also noticed that chemical doping directly with iodine vapors gives rise to highly doped conducting films only after a few seconds of exposition.^31^ In contrast, by exposing the OSC to an aqueous solution of the dopant, the diffusion of the dopant toward the OSC is slower and can be controlled, as previously demonstrated with an aqueous solution of Hg^2+^.^32^ Further, for the solvent annealing experiments, different polar solvents orthogonal to the OSC film were tested, but the best results were achieved when acetonitrile was used (Figure S1).

All the C8-BTBT-C8:PS thin films were inspected by polarized optical microscopy. The thin films exhibit a polycrystalline structure with large crystallites that did not undergo any visible change after performing the thin film treatments (Figure S2). The X-ray diffractograms of the films were also similar independently of the treatment procedure (see Figure S3). The appearance of the (*00l*) peaks is indicative of the formation of oriented crystals.^11,33^ Previous results already showed that the I_2_/water treatment did not affect the crystallinity of the films.^26^ We also investigated the X-ray reflectivity (XRR) profiles of C8-BTBT-C8:PS thin film samples before and after the treatment with acetonitrile and I_2_/CH_3_CN vapors (Figure S4). The fitting of the Kiessig fringes observed in the reflectivity curves was done to extract structural and morphological information with a simple layer model. A close look at the XRR curves reveals a slight reduction of the film thickness but a considerably rougher surface morphology after both vapor treatments (Table S1). Nevertheless, no prominent change in the crystal structure of C8-BTBT-C8 was observed in the treated samples. However, the XRR curves of the films after solvent vapor annealing show a reduction of the peak width, possibly due to the increasing size of the crystallites in the vertical direction.

The comparison of the UV–vis–NIR spectra of the pristine and treated films did not show any significant difference (Figure S5). The absence of a charge transfer absorption band is in agreement with the fact that no charge transfer process occurs between the C8-BTBT-C8 and iodine.^26^ UV resonance Raman spectra on pristine C8-BTBT-C8:PS film and on films doped with I_2_/water and I_2_/CH_3_CN vapors were performed. The use of a deep UV excitation wavelength of 266 nm ensures the resonance, which enhances the usual poor Raman scattering intensity from nanometric thick layers. This can be particularly interesting as the strong absorption of the excitation wavelength by the OSC allows for the detection of the very few monolayers from the doped surface. It should be noticed that UV–vis–NIR absorption, working in transmission configuration, probe both the doped surface and the deeper OSC layers, which could be much less affected by the doping, masking the effects of the iodine by averaging the signal over the entire film.^32^ UV resonance Raman showed no changes or vibrational modes shift in the spectral pattern comparing the pristine C8-BTBT-C8 with the doped films in the different experimental conditions (Figure S5), which confirms the absence of charge transfer complex or other chemical species formation.

**Figure 2:**
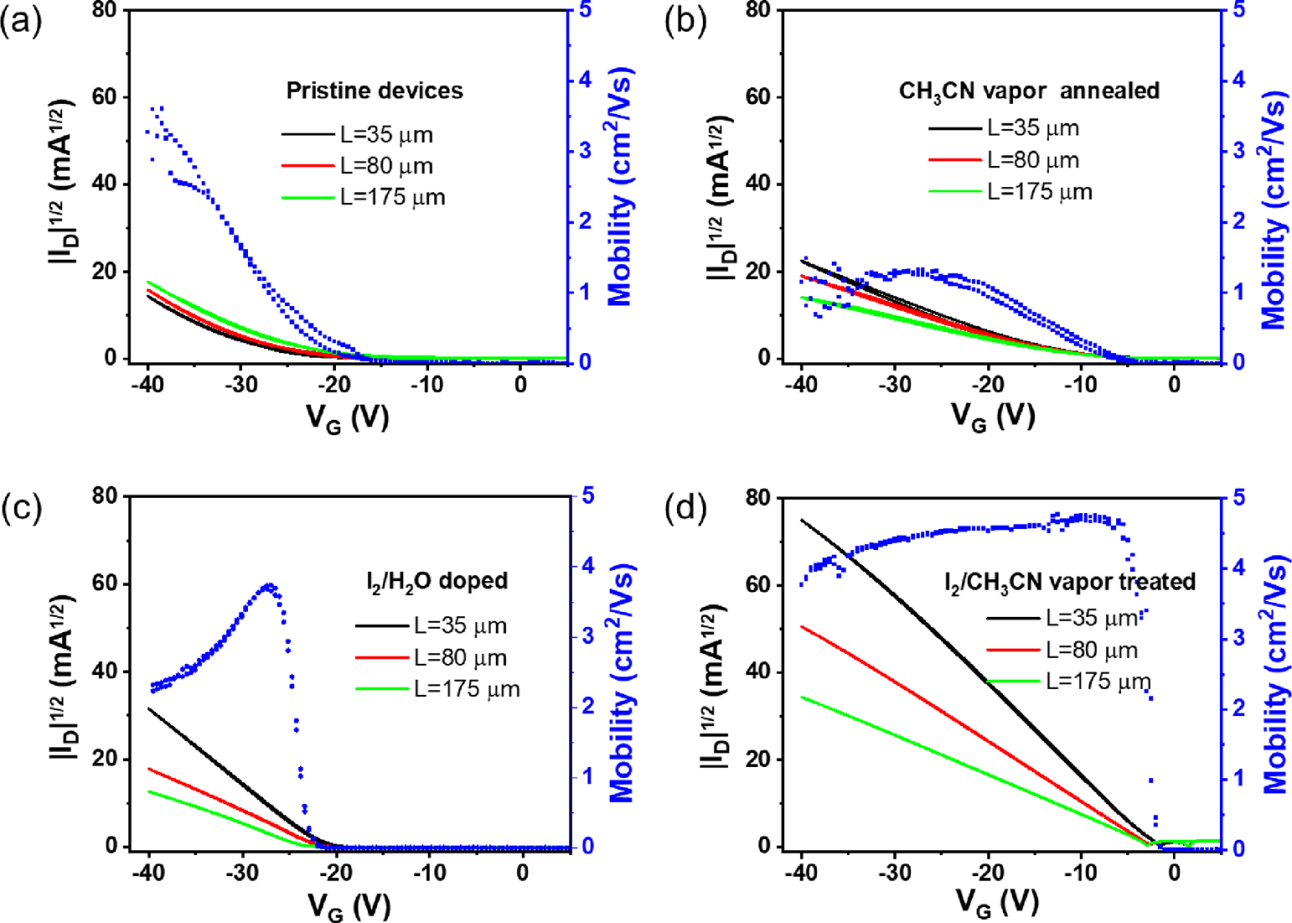
Square root of the absolute drain current of different channel lengths (*L*_1_ = 35 μm, *L*_2_ = 80 μm, and *L*_3_ = 175 μm) and charge carrier mobility (scattered curves) calculated from the transfer characteristics measured at a drain voltage of −40 V (forward and reverse) as a function of the gate voltage (*L*_3_ = 175 μm.): (a) pristine devices, (b) vapor annealed devices with CH_3_CN, (c) devices doped by I_2_/H_2_O, and (d) devices annealed and doped with I_2_/CH_3_CN vapors.

The OFET transfer characteristics of the devices were then investigated (Figure 2 and Figures S6 and S7). The corresponding output characteristics are shown in Figure S8. The device saturation mobility profile as a function of the gate voltage are also plotted in Figure 2 (for *L* = 175 μm). Pristine devices exhibit a nonideal curve shape and high-negative threshold voltage, and the current intensity is visibly not inversely scaling with L, which is a clear indication of high contact resistance. Further, the mobility shows a strong dependence with the gate voltage. After the CH_3_CN solvent annealing process, the device characteristics were improved, as noticed by a lower threshold voltage, transfer plots with source-drain current proportional with the different *L* values, and mobility less dependent on the gate voltage. An important current increase was observed when the devices were doped with I_2_/water, and additionally, the measured current also became inversely proportional to *L*. However, the mobility profile of these devices suffers from gate voltage dependency and a pronounced mobility peak is observed. Finally, the optimum device performance was achieved when the OSC films were exposed to vapors of I_2_/CH_3_CN. A low threshold voltage accompanied by a very high source-drain current and almost a gate-independent mobility was observed. The mobility values extracted in the saturation regime by standard fitting procedures for devices with different *L* values are illustrated in Figure S9. The average mobilities for pristine devices were 0.45, 1.27, and 1.68 cm^2^/Vs for devices with *L* = 35, 80, and 175 μm, respectively. The vapor-annealed films showed a lower average mobility of 1.02 cm^2^/Vs for the larger-channel devices, but, noteworthy, it was similar for all the *L*’s. Finally, the devices treated with I_2_/water and I_2_/CH_3_CN vapors showed increased mobilities of 2.55 and 4.11 cm^2^/Vs, respectively, and were *L*-independent.

In order to rationalize the results, we assessed the interfacial charge trap density (*N*_t_) of all these devices following two methodologies that provide slightly different information as detailed below. The first method relies on using the threshold voltage (*V*_th_) since it is the gate voltage required to fill traps at the OSC/dielectric interface before mobile charge carriers are accumulated.^34^ Thus, the trapped charges filled per unit area can be estimated using the following equation:^34^
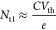
where *C* is the capacitance per unit area of the dielectric and *e* is the elementary charge.

An alternative common method to calculate *N*_t_ is employing the subthreshold swing (SS) according to^34^
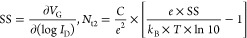
where *k*_B_ is the Boltzmann constant, *C* is the capacitance per unit area of the dielectric, *T* the absolute temperature, and *V*_G_ and *I*_D_ are the applied gate voltage and the measured drain current, respectively. In this expression, *N*_t2_ is the interfacial trap density per unit area per unit energy.

As mentioned, the distance of the trap energy states with respect to the band edge determines if they are shallow or deeper traps. Since the subthreshold region is defined by *V*_G_ < *V*_th_, the quasi-Fermi level is located far from the band edges (more than a few *K*_B_*T* from the HOMO level), and hence, the method based on the SS value probes deeper band gap states than the first one.^17^ In contrast, when the applied *V*_G_ increases and equals *V*_th_, the quasi-Fermi level is closer to the band edge, probing shallower interfacial traps. Therefore, *N*_t1_ will give an indication of shallow states, whereas *N*_t2_ will provide an estimation of deeper traps in the gap.

**Figure 3:**
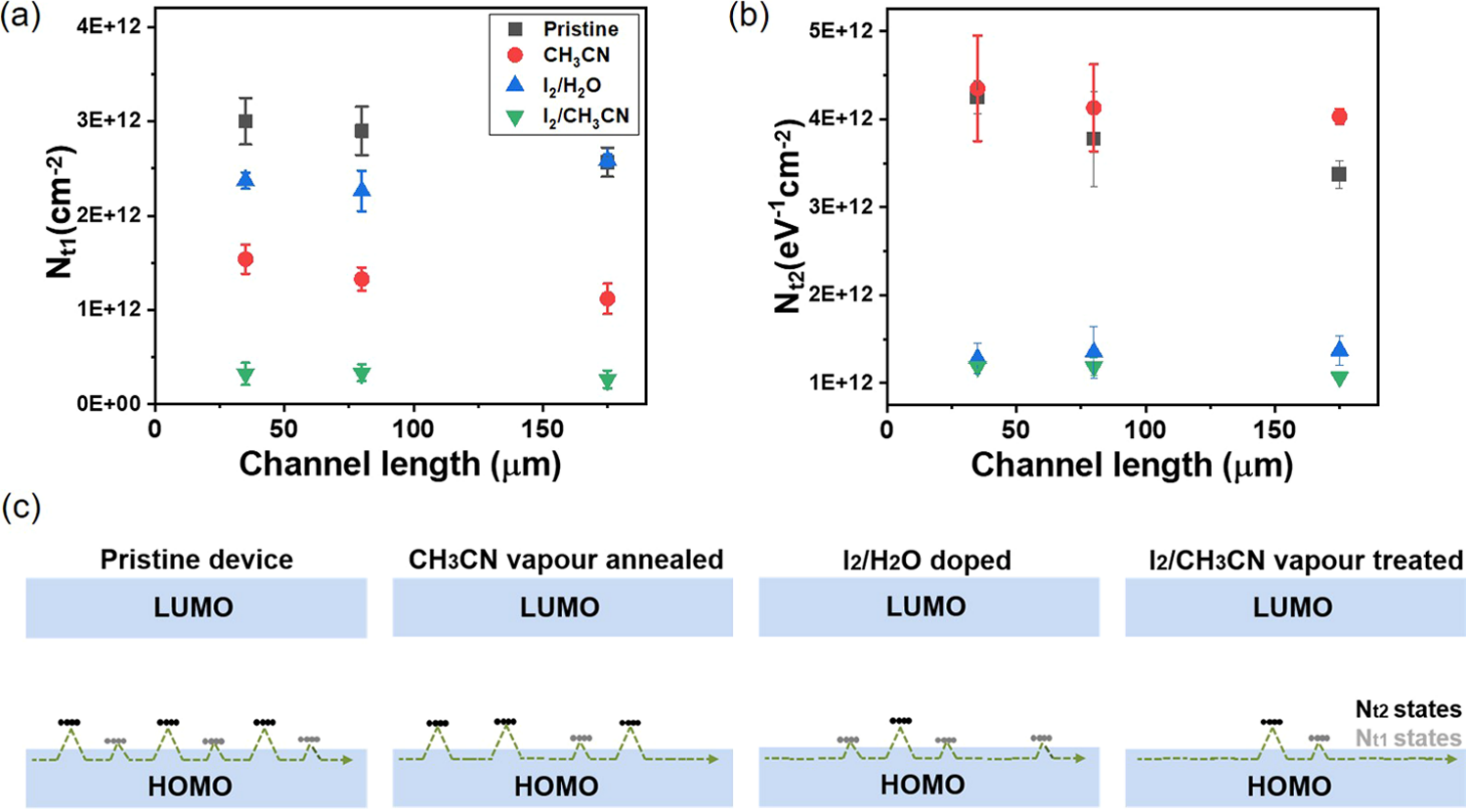
Shallow (*N*_t1_) and deep (*N*_t2_) trap estimation using the threshold voltage (a) and (b) subthreshold swing. (c) Schematic diagrams of the trap distribution in the devices.

Figure 3a,b presents the calculated *N*_t_ values for all the films using the two methods. It is observed that the OSC film treatment with I_2_/water only slightly reduces *N*_t1_. However, the solvent vapor annealing gives rise to an important reduction of this magnitude, which is also further diminished when the films are treated with vapors of I_2_/CH_3_CN. On the other hand, *N*_t2_ is strongly reduced in the films treated with both iodine-based doping procedures but is not affected (or even slightly increased) with the solvent vapor-annealed films. Thus, the solvent vapor annealing process seems to have a more significant impact on the shallow traps, while the iodine dopant is affecting the deeper traps. The optimum scenario is found with the films treated with vapors of I_2_/CH_3_CN since a synergistic effect is encountered. All these different situations are illustrated in Figure 3c.

**Figure 4:**
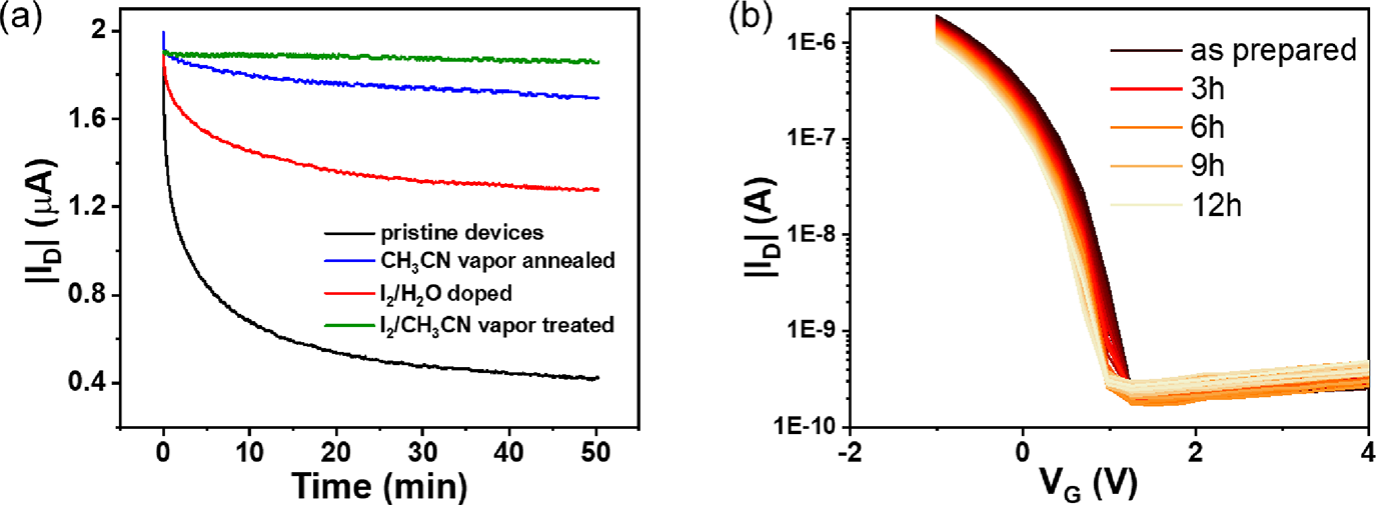
(a) Time-dependent drain current characteristics of pristine and treated devices (*V*_D_= −5 V). (b) Bias stress stability measurements of devices treated with I_2_/CH_3_CN vapors (*V*_G_ = −2 V, *V*_D_ = −5 V).

Bias stress stability measurements were conducted in order to evaluate the impact of the charge traps. Typically, under bias stress, a decrease in the source-drain current or a threshold voltage shift is observed, which is attributed to the entrapment of mobile charge carriers in localized electronic states.^35^Figure 4a shows the source-drain current monitored over 50 min for all the devices measured under ambient conditions. Notice that the same drain voltage (*V*_D_ = −5 V) was applied in all the cases, although different gate voltages were used to ensure a similar initial drain current (*I*_D_ ≈ 1.9 μA). It was found that the pristine devices exhibited an exponential current decrease with time. The devices based on films treated with acetonitrile vapors showed a much lower current decrease followed by the devices exposed to I_2_/water. Again, the most improved properties were found in the films treated with I_2_/CH_3_CN, which showed almost a constant current. Bias stress tests performing consecutive transfer plots (every 15 min for 12 h) were also performed (Figure 4b, Figure S10). Remarkably, the devices based on I_2_/CH_3_CN treated films showed overlapping transfer curves, a clear indication of their high stability and low level of traps. Only when a higher gate voltage was applied, the effects of the bias stress on the threshold voltage shift were more noticeable (Figure S11).

The existence of trap (localized) states in the OSC energy gap might also alter the effectiveness of carrier injection/collection, commonly visualized as a change in the device contact resistance, *R*_c_, a crucial parameter that can affect the device performance. We first calculated *R*_c_ using the standard transfer line method (TLM) (Figure S12).^36^ However, due to the nonideal device electrical characteristics, this method could not be successfully applied for the pristine films. The *R*_c_ values (at *V*_G_ = −40 V) were estimated to be 9000 Ω·cm for the CH_3_CN vapor annealed films, 1400 Ω·cm for the films exposed to I_2_/water, and 480 Ω·cm for the devices treated with I_2_/CH_3_CN vapors. This dramatic decrease in *R*_c_ corresponds to an 85% and 95% for the devices treated with I_2_/water and I_2_/CH_3_CN, respectively, in relation to the vapor annealed films.

**Figure 5:**
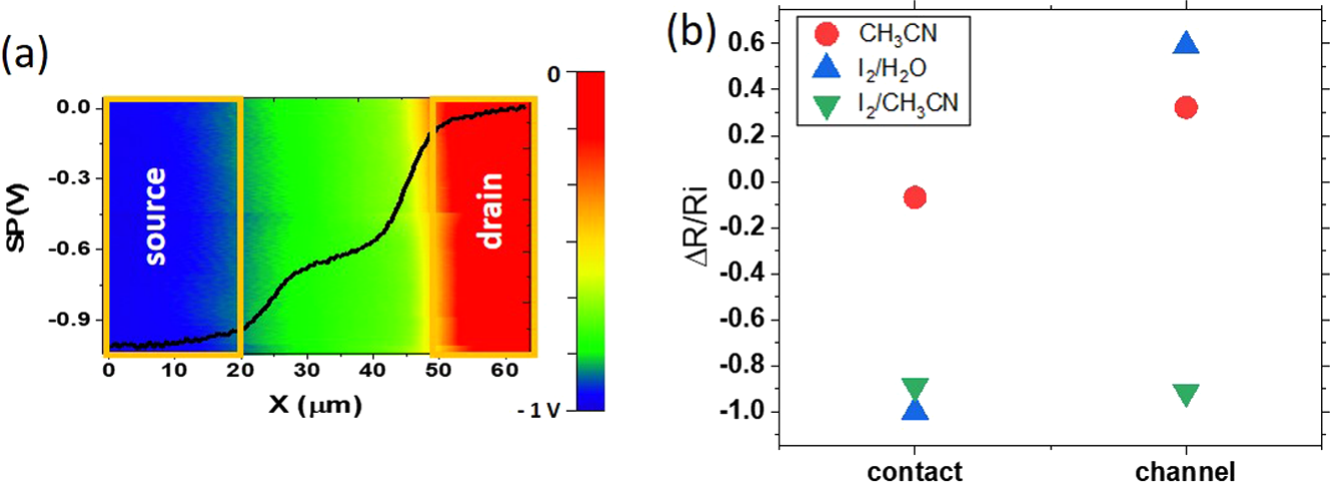
(a) Contact potential difference map measured by KPFM across electrodes and channel of a device after exposition to CH_3_CN vapor, data were taken under operation (*V*_D_ = −1 V, *V*_G_ = −40 V). A line profile from the image has been superimposed to illustrate the procedure for extracting the contact and channel resistances. Source and drain positions are indicated. (b) Relative values (Δ*R*/*R*_*i*_) of the contact resistance (*R*_c_ = *R*_d_ + *R*_s_) and channel resistance (*R*_ch_) with regard to the respective values for the pristine devices. The absolute values of the different resistances and typical contact potential KPFM profiles for the diverse devices are given in Figures S11 and S12 of the Supporting Information.

In order to further gain insights into the influence of the different treatments on *R*_c_, we employed scanning Kelvin probe force microscopy (KPFM) under operation, i.e., simultaneously measuring the current (*I*_D_) through the device. In such conditions, KPFM provides straightforward access to the actual potential drop across the whole device, at each electrode (i.e., source and drain), and at the channel. Hence, these local measurements allow obtaining the separated contributions of the contact resistance at drain (*R*_d_), source (*R*_s_), and channel (*R*_ch_). To tackle the unattainable statistics of local KPFM while accounting for any variability from device to device (e.g., deviations in initial performance), the measurements were carried out for the same device before (pristine) and after each treatment. To measure the contact resistance at both electrodes, the device with the short channel length was selected. Typical surface potential profiles taken along the channel and resistance values for the diverse devices are shown in the Supporting Information (Figures S13 and S14). The three pristine reference devices exhibit a larger value of *R*_c_ (i.e., *R*_d_ + *R*_s_) compared with *R*_ch_ (see Figure S13), herewith confirming that the nonideality of the devices is caused by the contact resistance. For the sake of comparison, the effect produced by each treatment is represented in Figure 5b as Δ*R*/*R_i_*, i.e. the change in resistance (*R*_c_ or *R*_ch_) relative to the respective value for the pristine devices (i.e., reference devices). It is observed that *R*_c_ is only reduced after the iodine-based doping procedures. As commented above, the contact resistance is a manifestation of the injection and collection barriers at the OSC/electrode interface and, therefore, is influenced as well by charge carrier trapping. Remarkably, a decrease by 90% in *R*_c_ for both types of iodine doping (i.e., I_2_/water and I_2_/CH_3_CN) with respect to the device treated with CH_3_CN vapor is in excellent agreement with the reduction obtained from standard TLM. Considering the estimation of the deep/shallow traps density (Figure 3), it is plausible to suggest that chemical doping primarily reduces deep traps at the contact interfaces. We observe that the beneficial effect of I_2_/water doping (blue symbols) is only reflected in the reduction of *R*_c_, while the dramatic decrease in both *R*_c_ and *R*_ch_ after the I_2_/CH_3_CN treatment (green symbols) is in consistency with the low level of shallow and deep traps evaluated after this treatment (Figure 3a,b).

Electronic traps in OSCs are a complex phenomenon that can be caused from varying factors.^17^ Whereas structural defects in the film are common sources of charge trapping, charge trapping sites are also typically found at the device interfaces, which are related to a large variety of effects (chemical impurities, local morphology and structure variations, energetic disorder, etc.).^37−42^ Although clarifying the sources of traps is a complex matter, the device improvements observed and rationalized in this work by the different treatments provide a better understanding of prevailing charge trapping mechanisms. We have observed that the vapor annealing of the films employing a polar solvent in which the OSC is not very soluble has given rise to a reduction of the shallow charge traps. X-ray characterization indicates that the crystallinity of the films is hardly altered by this treatment, exhibiting only a possible slight increase of the crystal size in the vertical direction. Recently, a solvent vapor annealing method was proposed to remove water-induced traps in OSCs.^28^ The authors stated that the annealing compacts the OSC layer, removing nanovoids and preventing the re-uptake of water. Although the origin of the traps that are removed by vapor annealing cannot be unambiguously identified, it is plausible that solvent vapor annealing leads to the healing of structural defects in the film and, herewith, to a reduction of low-energy traps. On the other hand, our results indicate that observed amelioration of charge injection upon treatment of the films with both iodine-based methods (possibly caused by a reduction of the energy barrier at the metal–OSC interface) is related to the filling of deeper charge carrier traps at the OSC–electrode interfaces. Remarkably, the synergistic effects found exposing the devices with I_2_/CH_3_CN vapors provide high-performance devices with ideal characteristics. In addition, these devices show not only high bias stress stability but also high time stability. As shown in Figure S15, after 5 weeks of storing the devices in environmental conditions, the saturation mobility only decreased from 4.70 to 3.13 cm^2^/Vs and the threshold voltage kept constant for all this time period. It should be also mentioned that this doping–annealing methodology has been proved to be successful for other BTBT derivatives, as for instance for the prototype semiconductor 2-decyl-7-phenyl-[1]benzothieno[3,2-*b*][1]benzo-thiophene (Ph-BTBT-10) (Figure S16).

## 3 Conclusions

In summary, we have fabricated OFETs based on thin films of C8-BTBT-C8:PS and we subjected them to different treatments with the aim at controlling and improving the device electrical characteristics. It was found that the CH_3_CN vapor annealing of the films does not affect the thin film structure, but this process is able to heal shallow interfacial traps. As a result, the corresponding OFETs show a lower threshold voltage and a mobility less dependent on the gate voltage. Further, the doping of the films by exposing them to a solution of I_2_/water results in a decrease of deeper interfacial traps and a significant reduction of the device contact resistance, giving OFETs with a higher mobility but with gate dependence. Finally, treating the films with vapors of I_2_/CH_3_CN successfully combines the solvent vapor annealing and the doping procedures. Here, devices with ideal characteristics are realized: low level of shallow/deep traps, optimized device mobility almost gate independent, high bias stress stability, and improved contact and channel resistance.

This work contributes toward the search for novel doping protocols that can improve the device performance and also brings new insights of the impact of the different OSC treatments on the thin film electrical properties. The findings here-reported represent a step forward toward the realization of trap-free organic semiconductor thin films with negligible *R*_c_ and optimized electrical performance, crucial for the progress of OFETs for practical applications.

## 4 Experimental Section

### 4.1 Device Preparation

C8-BTBT-C8 and polystyrene (PS, *M*_w_ = 10,000 g·mol^–1^) were purchased from Sigma–Aldrich and used without further purification. A solution of C8-BTBT-C8 and PS in chlorobenzene 2 wt % were prepared at a weight ratio of 4:1. C8-BTBT-C8:PS thin films were prepared by the bar-assisted meniscus shearing (BAMS) technique in ambient conditions on Si/SiO_2_ substrates (Si-Mat, SiO_2_ thickness is 200 nm, *C* = 17.26·10^–9^ F·cm^–2^).^3,29^ Previously, to the deposition of the OSC solution, the substrates were cleaned with acetone and isopropanol 3 times each and then dried under a nitrogen flow. Top source-drain gold contacts (25 nm) were deposited by thermal evaporation through a shadow mask with channel width *W* = 4 mm and channel lengths of *L* = 50, 100, and 200 μm. After the contacts evaporation, samples were kept in dark in air 7 days.

### 4.2 OSC Treatments

Iodine solid and acetonitrile were purchased from Sigma-Aldrich and used directly.

#### 4.2.1 Acetonitrile Vapor Annealing

6 mL of CH_3_CN were placed in a glass container with a diameter of 2.2 cm and a height of 3.0 cm. Then, the substrates were fixed on a Petri dish and used to cover the container. The films were exposed to the solution vapors for 10 min.

#### 4.2.2 I_2_/Water Treatment

Doping treatment was done by exposing the top surface of the devices to an aqueous saturated iodine solution. A droplet of the solution was casted on the device, completely covering the OFET channel. After 3 min, the device was abundantly washed with MilliQ water and dried with a nitrogen flow*.*

#### 4.2.3 I_2_/CH_3_CN Treatment

A 0.17 mg/mL iodine solution in CH_3_CN was prepared (6 mL in a glass container with a diameter of 2.2 cm and a height of 3 cm). Following the same methodology as in 1), the films were exposed to the vapors of the solution for 10 min.

### 4.3 Electrical Measurements

Electrical measurements were performed in ambient conditions employing a two channel Keithley 2612 source meter. The bias stress stability measurements were done using an Agilent B1500A semiconductor devices analyzer connected to the samples with a Karl Suss probe station. Transfer characteristic were measured in the linear and saturation regime, swept forward and reverse. The mobility values were extracted in the saturation regime according to
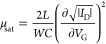


The threshold voltage (*V*_th_) was extracted using the following equation:
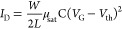
where *C* is the insulator capacitance per unit area and *W* and *L* are the width and length of the channel, respectively.

### 4.4 X-ray Characterization

X-ray specular diffractograms in the 2θ range 2.5°–10° were collected on a Siemens D-5000 diffractometer. X-ray reflectivity (XRR) measurements were made using a Panalytical Empyrean reflectometer using Cu Kα radiation (wavelength λ = 0.15406 nm). On the primary side, a multilayer mirror was used to render the parallel beam; on the secondary part, a combined set up with a receiving slit, a Soller slit and a PANalytical PIXCEL3D detector was used. The 2θ (angular scans) have been recalculated to scattering vector notation using *qz* = 4π/λ × sin θ. XRR data were fitted using the software “X’Pert Reflectivity 1.3” (PANalytical, Netherlands), and surface roughness was characterized by the model of Névot & Croce.^43,44^

### 4.5 UV–vis–NIR Spectroscopy

The absorption spectra of the films were measured with a UV–visible-NIR spectrophotometer (V-780).

### 4.6 UV-Resonance Raman

Raman measurements were carried out at the IUVS beamline at Elettra Synchrotron radiation facility, in Trieste. The sample is excited with a 266 nm diode laser in backscattering configuration using a Czerny–Turner single stage *f* = 750 mm spectrometer (Trivista, Princeton Instruments) coupled with a Peltier cooled back-thinned CCD (Princeton Instruments). A detailed description of the experimental apparatus is reported elsewhere.^45^

### 4.7 Atomic force microscopy (AFM) and Kelvin probe force microscopy (KPFM)

AFM and KPFM data were acquired using a commercial head and control electronics from Nanotec Electronica. Conducting CrPt-coated Si tips mounted on rectangular cantilevers from BudgetSensors, with nominal force constant *k* = 3 N/m and 75 kHz of resonance frequency, were used. KPFM was employed in the frequency modulation (FM) mode to measure local surface contact potential differences (SP) on the devices under operation conditions. During FM-KPFM measurements, the tip is excited by an AC voltage (∼0.5 V) at a given frequency (*f*_ac_ ≈ 0.7 kHz) while a feedback loop adjusts the dc bias needed to nullify the frequency shift (Δ*f*) of the mechanical oscillation, which is proportional to the electrostatic force gradient. In our setup, the voltage is applied to the tip so that the higher the surface potential, the lower the work function (ϕ). SP maps are obtained simultaneously with topography in a single pass mode. The OFETs were mounted in the AFM and were operated and measured with two Keithley 2450 system source-meter instruments. All presented images were analyzed by using the WSxM freeware.^46^

## Supporting Information Available

Polarized optical microscopy and AFM images, X-ray diffractograms, spectroscopic characterization, and additional electrical measurements and data (PDF)
