# Supplementary material for: Synergistic Effect of Solvent Vapor Annealing and Chemical Doping for Achieving High-Performance Organic Field-Effect Transistors with Ideal Electrical Characteristics
Source: ACS Appl Mater Interfaces. 2023 Jan 18;15(4):5521–8. doi: 10.1021/acsami.2c16760 (PMC9949699; doi:10.1021/acsami.2c16760)
Supplement: Supplementary file 1 — am2c16760_si_001.pdf [file am2c16760_si_001.pdf]

Supporting Information

Synergistic Effect of Solvent Vapour Annealing and Chemical Doping for Achieving High Perfomance Organic Field-Effect Transistors with Ideal Electrical Characteristics

Jinghai Li,[1] Adara Babuji,[1] Lamiaa Fijahi,[1] Ann Maria James,[2] Roland Resel,[2] Tommaso Salzillo,[3] Raphael Pfattner,[1] Carmen Ocal,[1] Esther Barrena,[1*] Marta Mas-Torrent[1*]

[1] Institut de Ciència de Materials de Barcelona, ICMAB-CSIC, Campus UAB, 08193 Bellaterra, Spain. E-mail: ebarrena@icmab.es; mmas@icmab.es

[2] Institute of Solid State Physics, Graz University of Technology, Petersgasse 16, Graz 8010, Austria.

[3] Dipartimento di Chimica Industriale "Toso Montanari", University of Bologna, Viale del Risorgimento 4, 40136, Bologna, Italy.

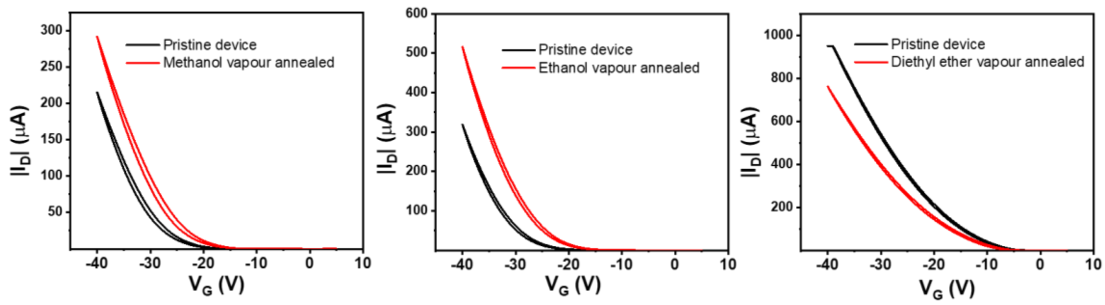

Figure S1. Transfer characteristics of C8-BTBT-C8:PS thin film OFETs before and after solvent annealed with vapors of methanol, ethanol and diethyl ether.

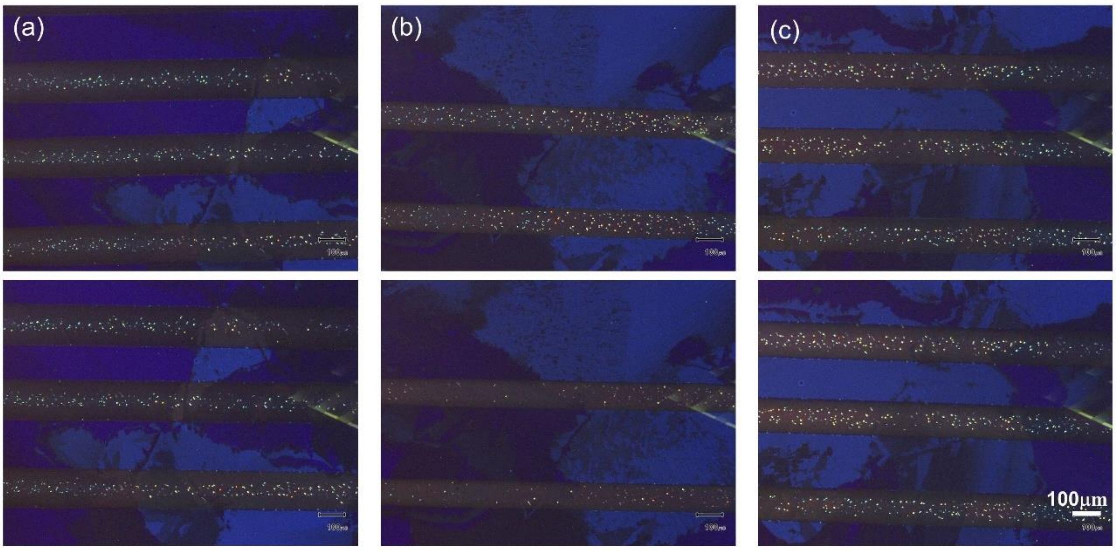

Figure S2. Polarized optical microscopy images of C8-BTBT-C8:PS thin films. The top images correspond to pristine films and the bottom ones are the same films after: (a) vapour annealed with CH_3CN, (b) doped by I_2/H_2O and (c) doped and annealed with I_2/CH_3CN vapours.

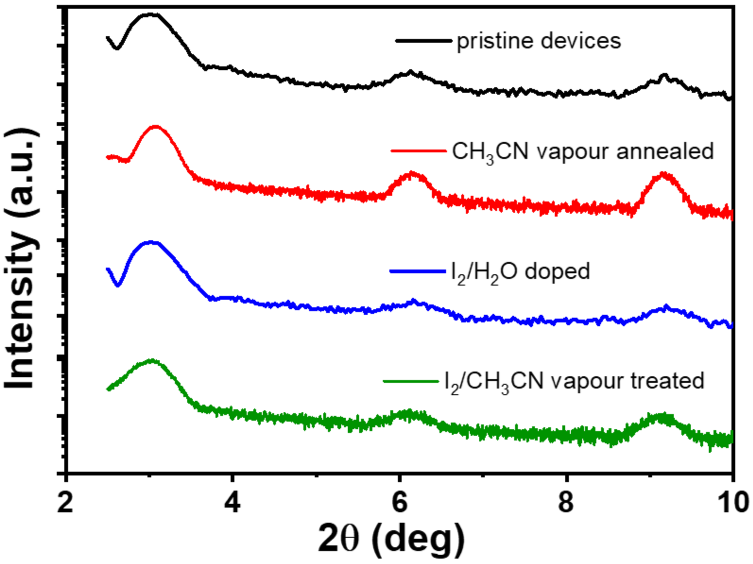

Figure S3. XRD of C8-BTBT-C8:PS films before and after the different treatments.

.

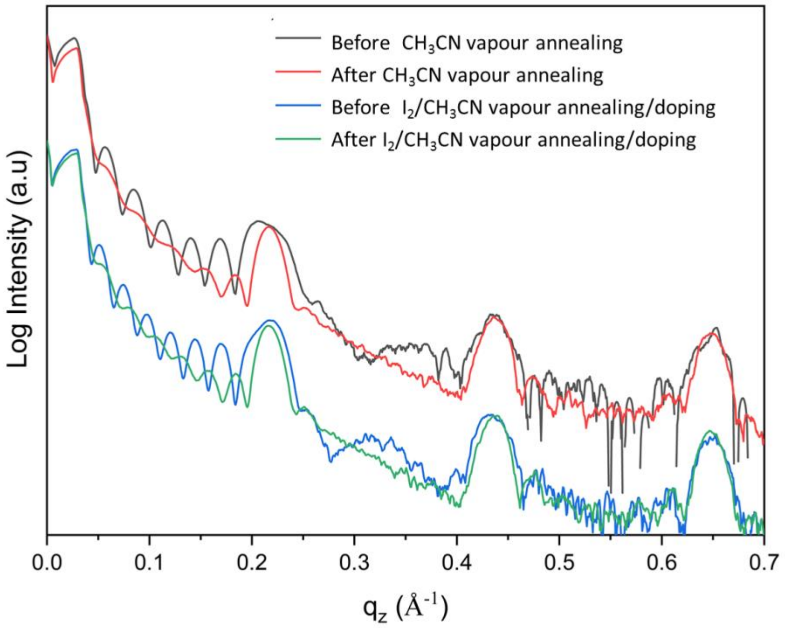

Figure S4. XRR diffractograms of pristine C8-BTBT-C8:PS films and the corresponding films after CH_3CN vapour annealing and I_2/CH_3CN vapour annealing and doping.

Table S1: Thin film morphology obtained from fitting of the X-ray reflectivity data in the region of the Kiessig fringes ($< q_z = 0.15$ Å^{-1}) revealing film thickness and surface roughness.

sample	thickness (nm)	roughness (nm)
before CH_3CN vapour annealing	22.6	0.4
after CH_3CN vapour annealing	21.5	3.3
before I_2/CH_3CN vapour annealing/doping	26.4	0.3
after I_2/CH_3CN vapour annealing/doping	23.3	2.5

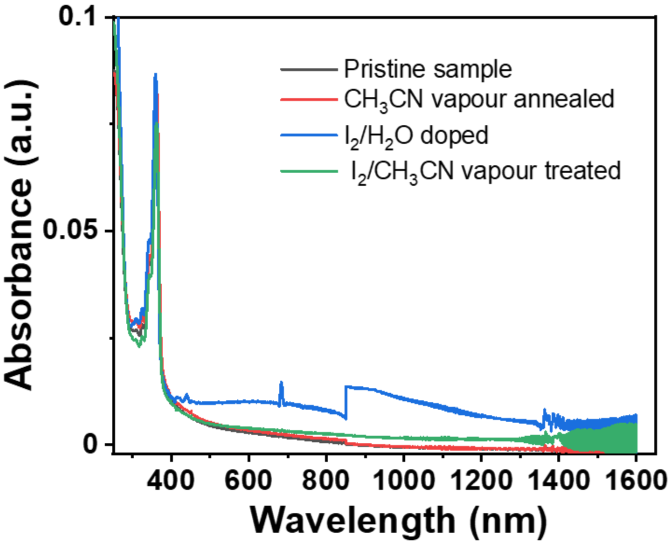

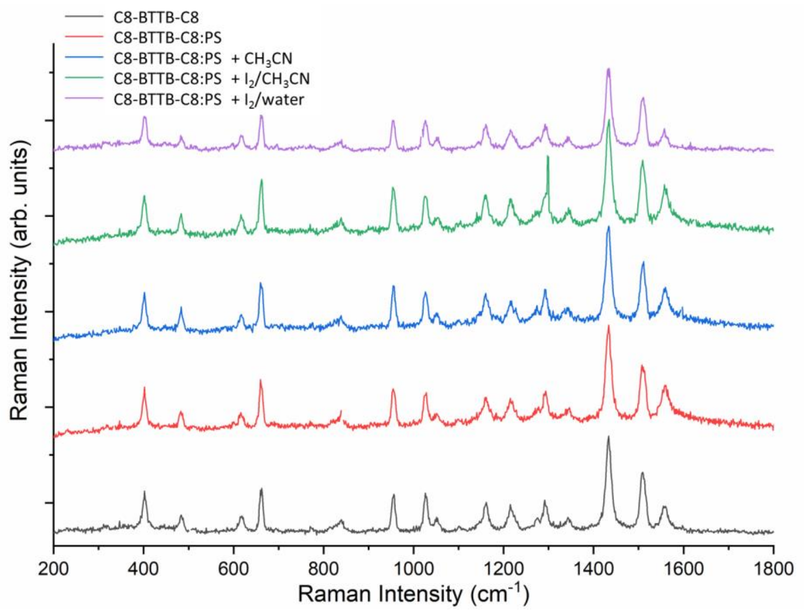

Figure S5. Top. UV-Vis of C8-BTTB-C8:PS films before and after CH_3CN and I_2/CH_3CN treatments. Bottom. UV Resonance Raman spectra of the C8-BTTB-C8 films before and after the different treatments.

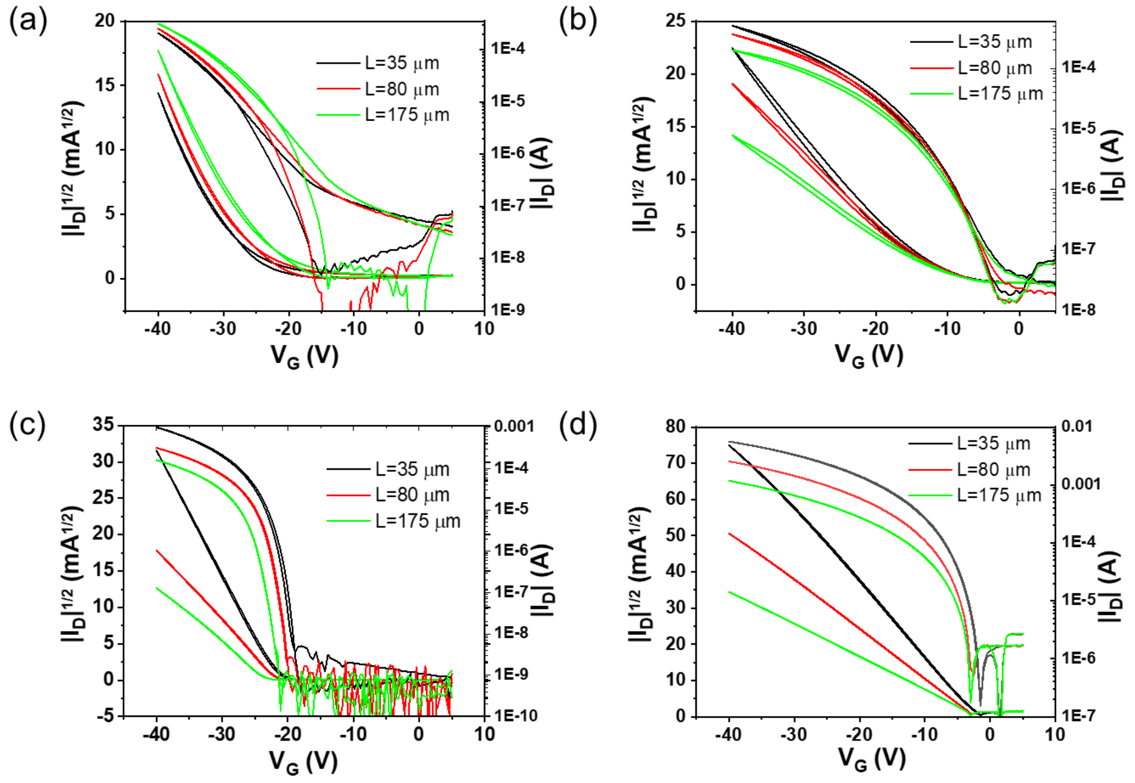

Figure S6. Saturation transfer characteristics (V_D= - 40 V) of different lengths. (a) pristine devices, (b) devices annealed with CH_3CN vapours, (c) devices doped by I_2/H_2O and (d) devices doped and annealed with I_2/CH_3CN vapours. The measurement settings for (d) had to be modified due to the high current of this device, affecting the sensitivity at lower currents.

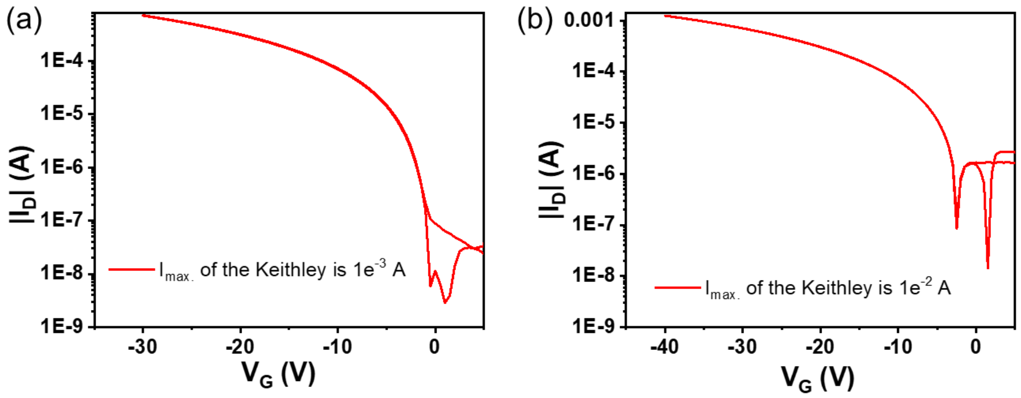

Figure S7. Saturation transfer characteristics (V_D= - 40 V) of the same OFET device after I_2/CH_3CN treated measured with different settings: (a) Maximum current value of the Keithley unit is set to $1 \cdot 10^{-3}$ A and (b) maximum current value of the Keithley unit is set to 10^{-2} A. As observed, by increasing the maximum measured current, we lose sensitivity of the off current, an effect that can be observed in the larger off current in Figure S6(d).

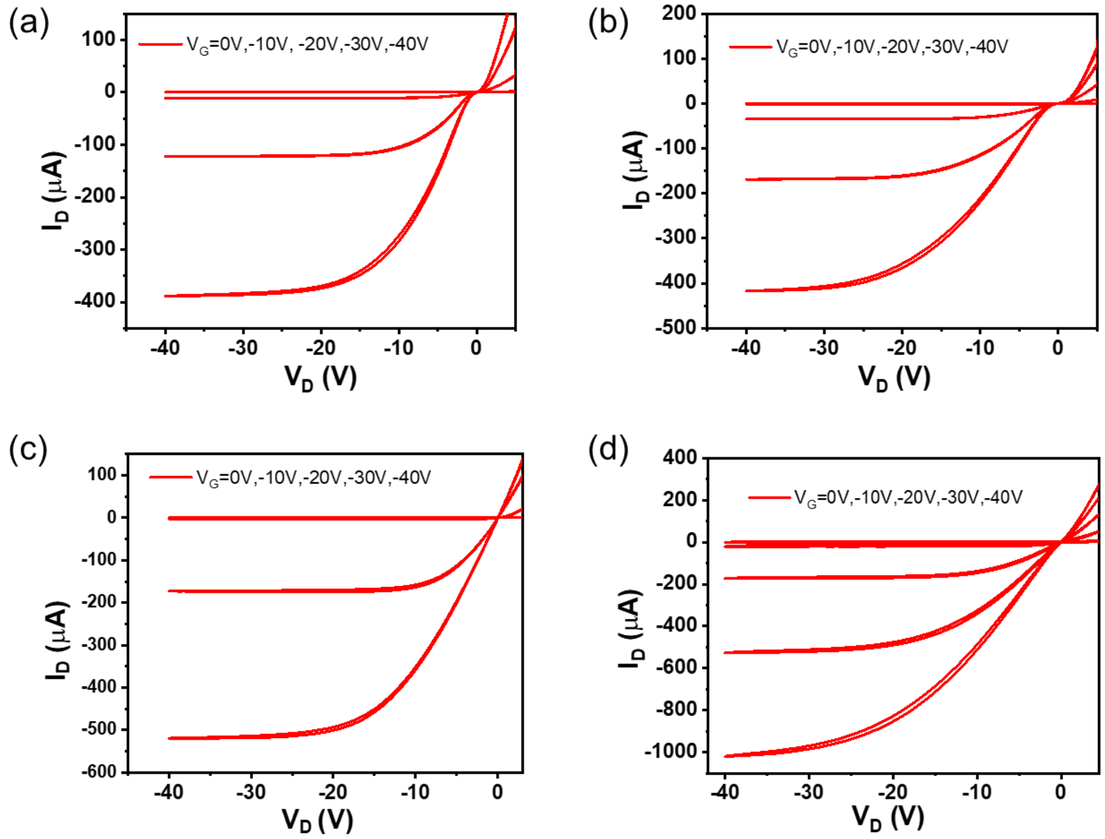

Figure S8. Output characteristics for OFETs with L= 80 μm. (a) pristine devices, (b) devices vapour annealed with CH_3CN, (c) devices doped by I_2/H_2O and (d) devices doped and annealed with I_2/CH_3CN vapours.

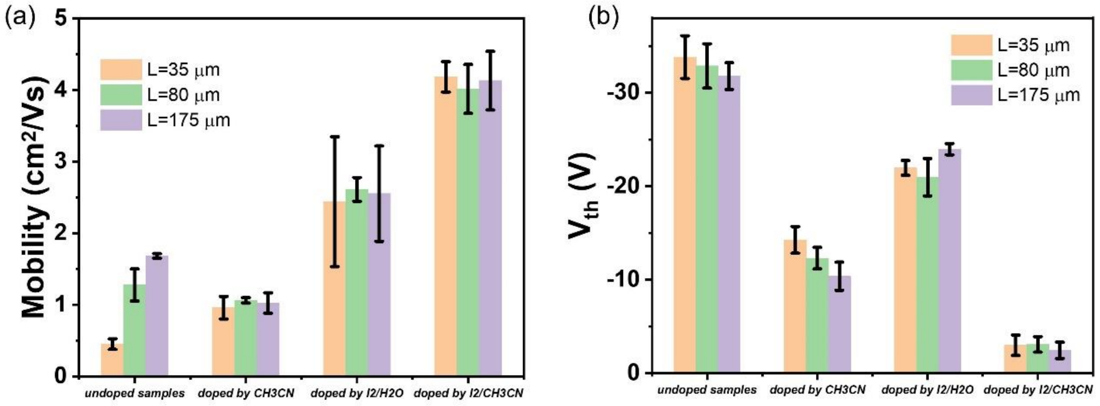

Figure S9. (a) Mobility and (b) threshold voltage of pristine and treated devices with different channel lengths. The error bars of the standard deviation are also included.

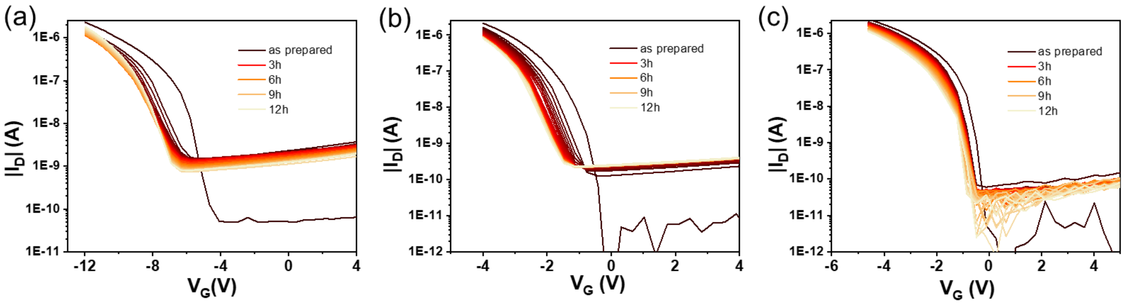

Figure S10. Bias stress stability measurements performed by applying V_D= -5 V and gate voltages giving similar source-drain current, and measuring the transfer characteristics (V_D= -5 V) every 15 min: (a) a pristine device (V_G = - 12 V), (b) a device annealed with CH_3CN vapour (V_G = - 4 V) and (c) a device doped with I_2/H_2O (V_G = - 4.5 V).

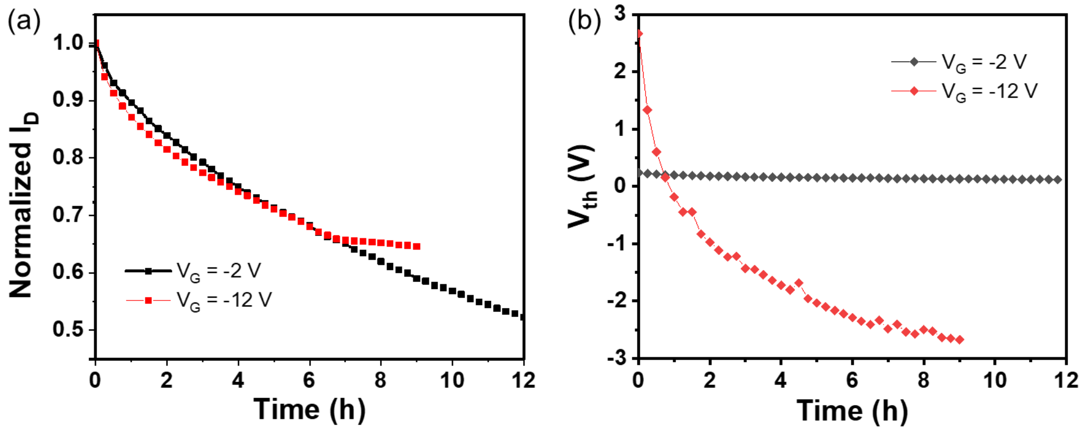

Figure S11. Comparison of the bias stress stability measurements performed to a device treated with I_2/ CH_3CN by applying a V_G= -12 and -2 V (V_D= -5 V). In all the devices, transfer characteristics were performed every 15 min. (a) Maximum I_D vs. time and (b) V_{th} vs. time.

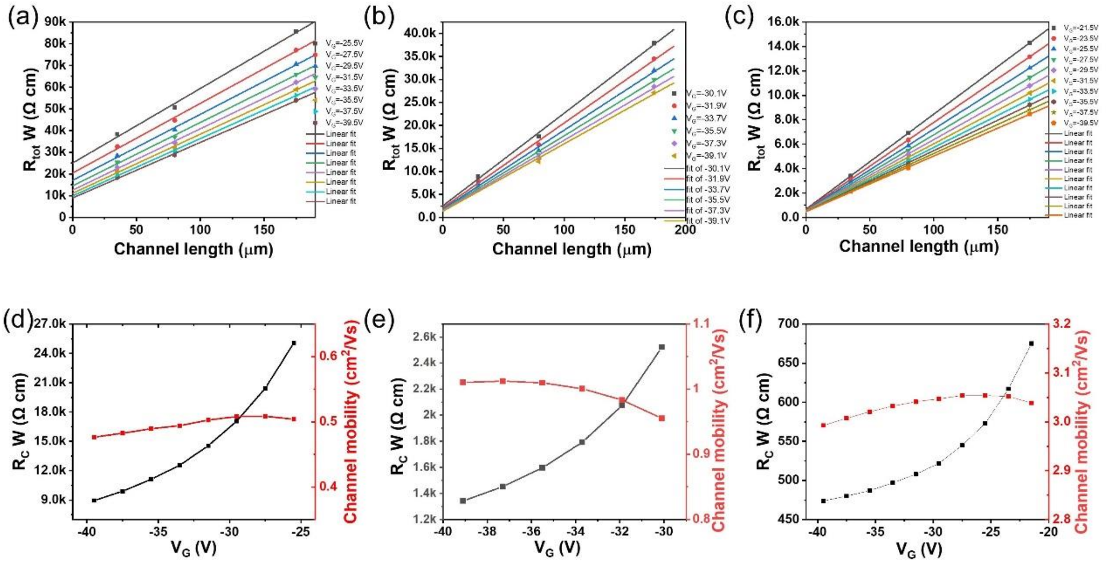

Figure S12. Transfer line method plot and mobility corrected by contact resistance and contact resistance as a function of gate voltage. (a) and (d) devices annealed with CH_3CN vapours, (b) and (e) devices doped with I_2/H_2O, (c) and (f) devices doped and annealed with I_2/CH_3CN vapours.

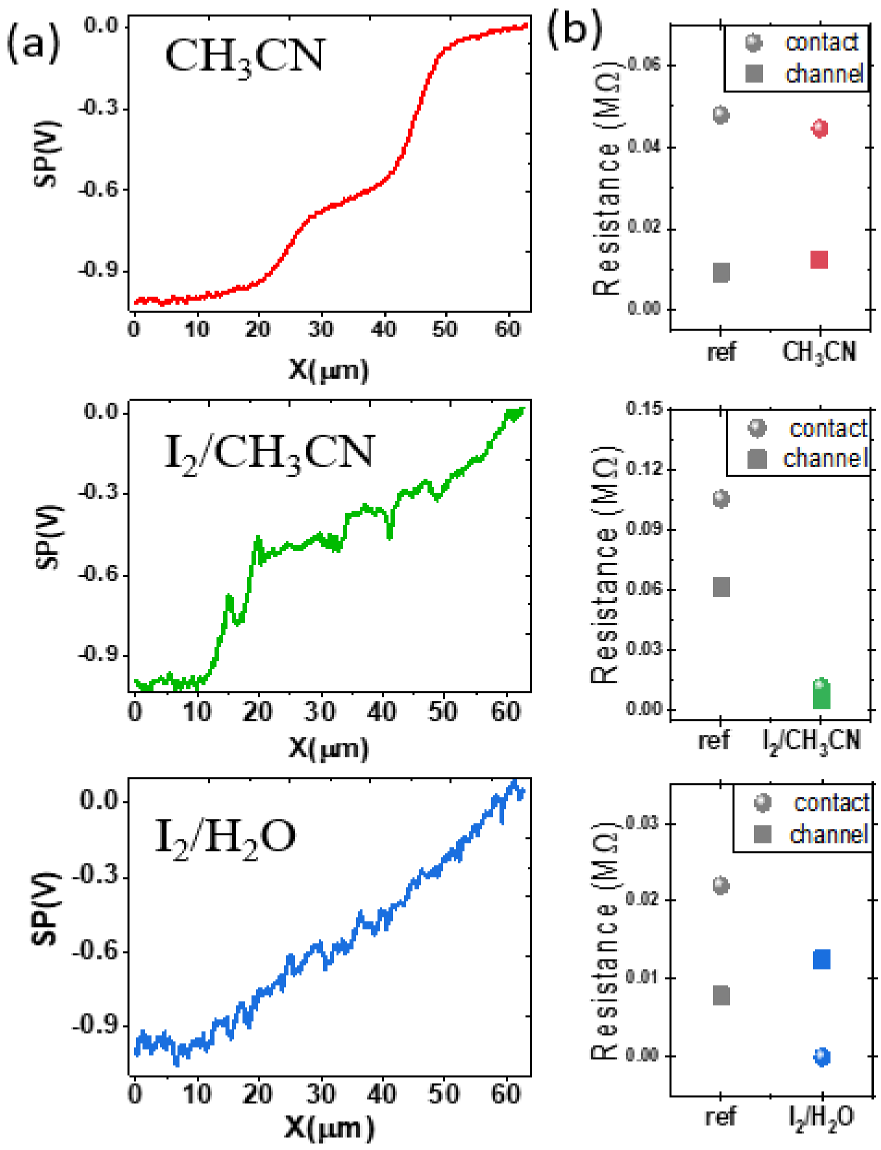

Figure S13. (a) Contact potential difference profiles measured by KPFM after the device treatment indicated. (b) Absolute resistance values of contact and channel for the respective pristine and treated devices. V_D= -1 V, V_G= -40 V.

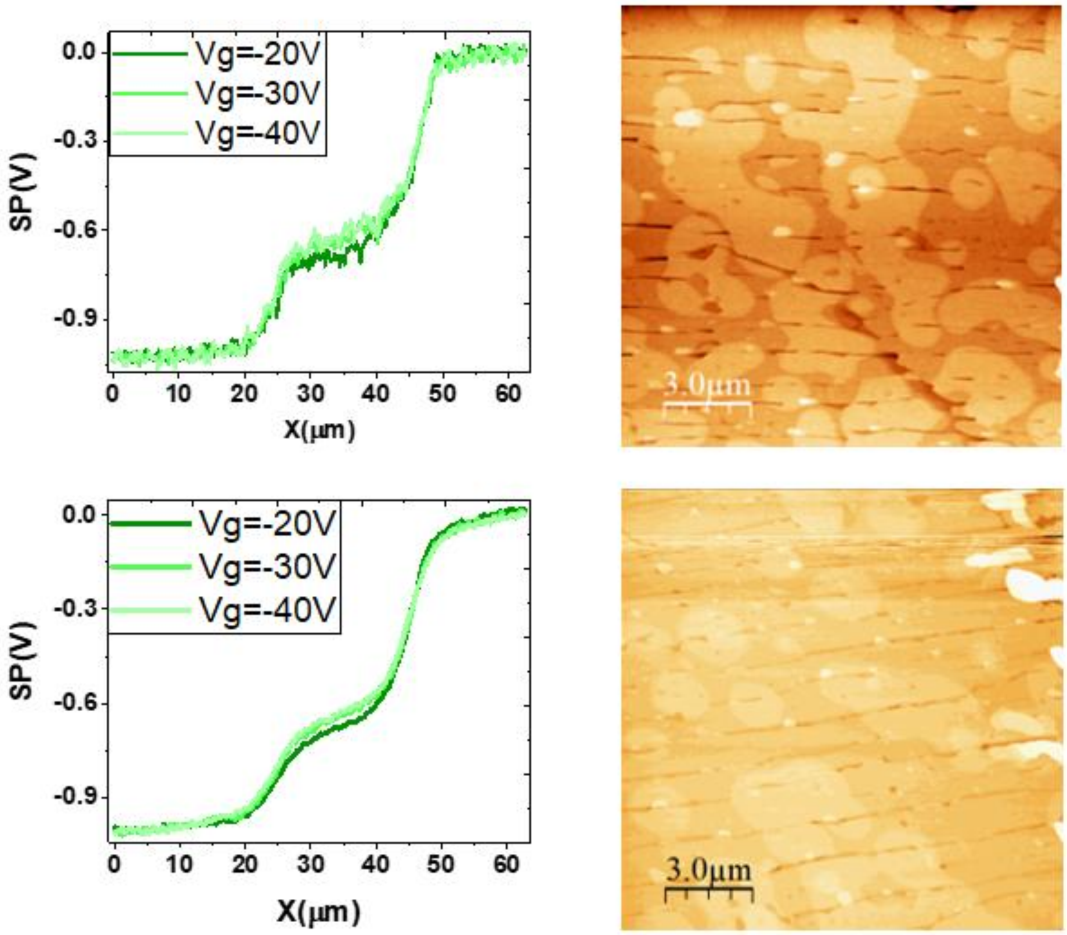

Figure S14. Left panels: Contact potential difference profiles measured by KPFM before (top) and after (bottom) I_2/CH_3CN doping. Right panels: Topographic AFM images obtained at the channels centre of the same devices before (top) and after (bottom) the doping treatment. The device was measured under operation at V_D= -1V and the indicated V_G.

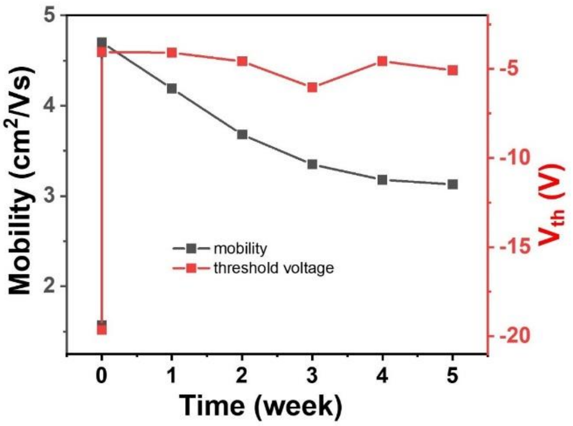

Figure S15. Mobility and threshold voltage evolution with time of devices exposed to I_2/CH_3CN vapours.

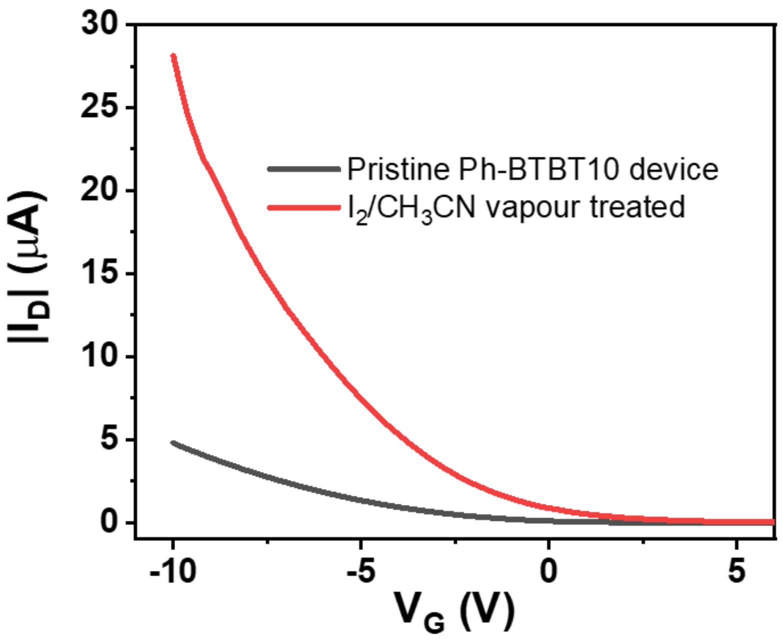

Figure S16. Transfer characteristics of a thin film based on Ph-BTBT-10 deposited by BAMS before and after treatment with I_2/CH_3CN vapours.
